# Interfering B cell receptor signaling via SHP-1/p-Lyn axis shows therapeutic potential in diffuse large B-cell lymphoma

**DOI:** 10.1186/s10020-022-00518-0

**Published:** 2022-08-08

**Authors:** Ji-Lin Chen, Pei-Yi Chu, Chun-Teng Huang, Tzu-Ting Huang, Wan-Lun Wang, Yu-Hsuan Lee, Yuan-Ya Chang, Ming-Shen Dai, Chung-Wai Shiau, Chun-Yu Liu

**Affiliations:** 1 Comprehensive Breast Health Center, Taipei Veterans General Hospital, No. 201, Sec. 2, Shih-Pai Road, Taipei, 112 Taiwan; 2 Department of Pathology, Show Chwan Memorial Hospital, No. 542, Sec. 1, Chung-Shan Rd., Changhua City, 500 Taiwan; 3 School of Medicine, Fu Jen Catholic University, No. 510, Zhong-zheng Rd., Xin-zhuang Dist., New Taipei City, 24205 Taiwan; 4 Department of Health Food, Chung Chou University of Science and Technology, Changhua, 510 Taiwan; 5 School of Medicine, National Yang Ming Chiao Tung University, Hsinchu, 30010 Taiwan; 6 Division of Hematology and Oncology, Department of Medicine, Yang-Ming Branch of Taipei City Hospital, No.145, Zhengzhou Rd., Datong Dist., Taipei, 10341 Taiwan; 7 Division of Medical Oncology, Department of Oncology, Taipei Veterans General Hospital, No. 201, Sec. 2, Shih-Pai Road, Taipei, 112 Taiwan; 8 Hematology/Oncology, Tri-Service General Hospital, National Defense Medical Center, Taipei, Taiwan; 9 Institute of Biopharmaceutical Sciences, National Yang Ming Chiao Tung University, No. 155, Sec. 2, Li-Nong Street, Taipei, 112 Taiwan; 10 Division of Transfusion Medicine, Department of Medicine, Taipei Veterans General Hospital, No. 201, Sec. 2, Shih-Pai Road, Taipei, 112 Taiwan

**Keywords:** Diffuse large B cell lymphoma, SHP-1, Apoptosis

## Abstract

**Background:**

Diffuse large B cell lymphoma (DLBCL) is an aggressive and molecularly heterogeneous non-Hodgkin’s lymphoma. The B cell receptor (BCR) signaling pathway in DLBCL emerges as a new drug target. Protein phosphatase SHP-1 negatively regulates several oncogenic tyrosine kinases and plays a tumor suppressive role.

**Methods:**

The direct SHP-1 agonists were used to evaluate the potential therapeutic implication of SHP-1 in DLBCL. Immunohistochemical staining for SHP-1 was quantified by H-score. The SHP-1 phosphatase activity was determined using tyrosine phosphatase assay. In vitro studies, including MTT, western blot analysis and cell apoptosis, were utilized to examined biological functions of SHP-1.

**Results:**

Oral administration of SHP-1 agonist showed the potent anti-tumor effects compared to a selective Bruton’s tyrosine kinase (BTK) inhibitor ibrutinib in mice bearing U2932 xenografts. SHP-1 agonist increased SHP-1 activity as well as downregulated p-Lyn in vivo. Here, we demonstrated that immunohistochemical staining for SHP-1 expression was positive in 76% of DLBCL samples. SHP-1 agonist exerted anti-proliferative and apoptotic effects compared with ibrutinib in DLBCL cells. Mechanistically, SHP-1 agonist decreased BCR signaling, especially p-Lyn, and led to apoptosis.

**Conclusions:**

These data suggest that SHP-1 negatively regulates phosphorylation of Lyn, and targeting SHP-1/p-Lyn using SHP-1 agonist has therapeutic potential for treatment of DLBCL.

**Supplementary Information:**

The online version contains supplementary material available at 10.1186/s10020-022-00518-0.

## Background

Diffuse large B cell lymphoma (DLBCL) is a common and aggressive form of non-Hodgkin lymphoma (NHL). Molecular profiling studies have revealed molecular heterogeneity in DLBCL tumors, classified the cell-of-origin into at least two distinct molecular subtypes: germinal center B-cell (GCB) and activated B-cell (ABC), with a generally worse clinical outcome for ABC-type DLBCL (Alizadeh et al. 2000; Hans et al. 2004; Visco et al. 2012). Despite most DLBCL tumors being initially sensitive to chemotherapy or radiotherapy, refractory/relapsed DLBCL confers a poor outcome underscoring the need for new therapeutic options (Camicia et al. 2015).

B cell receptor (BCR) survival signaling is emerging as an important therapeutic targeting pathway due to recent advances in inhibitors targeting key enzymes of the pathway, such as Bruton’s tyrosine kinase (BTK), spleen tyrosine kinase, mammalian target of rapamycin, and phosphoinositide 3'-kinase δ isoform (Wiestner 2015). The therapeutic potential of the BCR pathway in DLBCL has also been investigated (Wilson et al. 2015; Young et al. 2015). Ibrutinib, a selective BTK inhibitor, has been approved for mantle cell lymphoma, chronic lymphocytic leukemia, Waldenström's macroglobulinemia, and marginal zone lymphoma (Gayko et al. 2015) and has demonstrated in vitro and in vivo efficacy in DLBCL, underscoring the role of BCR signaling in DLBCL (Wilson et al. 2015; Ezell et al. 2014; Mathews Griner et al. 2014). Wilson et al. conducted a phase I/II trial of ibrutinib monotherapy that involved 80 patients with relapsed or refractory DLBCL and found ibrutinib produced tumor responses in 25% (20/80) of patients overall, and in 37% (14/38) of patients with ABC-type DLBCL, compared to 5% (1/20) of patients with GCB–like DLBCL (Wilson et al. 2015).

Lyn, as a member of the Src family of intracellular membrane-associated tyrosine kinases, is involved in signal transmission for a variety of receptors, including B-cell receptors (Ingley 2012). The phosphorylation at Tyr397 residue within the activation loop is critical for activation of the enzyme (Williams et al. 2009). Lyn appears to play a critical role in B cell receptor signaling due to its dual modulating capacity via activating or inhibiting downstream pathways (Ingley 2012). For example, a raft-associated signalosome made of the constitutively active Lyn kinase, the tyrosine phosphorylated Cbp/PAG adaptor, and tyrosine phosphorylated STAT3 transcription factor (the Lyn-Cbp/PAG signalosome), appears to control proliferation and survival in several B-NHLs cells including DLBCL cell lines SU-DHL-6, OCI-Ly3, and constitutes a therapeutic target in B-NHL cells that exhibit oncogenic “addiction” to the Lyn kinase (Tauzin et al. 2008). In contrast, reduced Lyn kinase activity in the context of CD79 mutations in ABC-type DLBCL may augment ongoing chronic active BCR signaling (Young et al. 2015). Nevertheless, the plethora functions of Lyn have made it an investigational therapeutic target of interest for several hematological as well as solid cancers (Ingley 2012). Interestingly, in the inactivation cycle of Lyn, protein tyrosine phosphatases such as Src homology region 2 domain containing phosphatase 1 (SHP-1) are known to dephosphorylate the activation loop site, and are thus important in down-regulating Lyn (Samanta et al. 2009; Somani et al. 2001).

The tumor suppressive role of SHP-1, encoded by *PTPN6* gene, has been made clear by reports of its negative regulation of several key oncogenic tyrosine kinases such as the JAK kinases (David et al. 1995; Jiao et al. 1996; Haque et al. 1998; Migone et al. 1998) and STAT3 (Tai et al. 2011). SHP-1-mediated STAT3-inhibition and subsequent apoptosis induction is an appealing anti-cancer strategy that has been reviewed (Huang et al. 2017). The therapeutic potential of SHP-1 can be strengthened by a generation of SHP-1 activity enhancers, including sorafenib derivatives such as SC-43 and SC-60. SC-43 interacts with the inhibitory N-SH2 domain of SHP-1 leading to SHP-1 activation (Su et al. 2017). These sorafenib derivatives have been shown to directly increase SHP-1 activity and have been tested in a variety of solid cancer cells and clinical trial (NCT04733521) (Liu et al. 2017a; Chung et al. 2018). In this study, we examined the role and potential therapeutic implication of SHP-1/p-Lyn signaling in DLBCL. We highlight the importance of the axis formed by the tumor suppressor SHP-1 and the oncogenic p-Lyn in DLBCL.

## Methods

### Cell culture and transfection

The DLBCL cell lines, DB cells were obtained from the American Type Culture Collection (Manassas, VA, USA). U2932, SU-DHL-6, and OCI-Ly7 cells were purchased from Deutsche Sammlung von Mikroorganismen und Zellkulturen (Braunschweig, Germany). U2932, SU-DHL-6 and DB cells were maintained in RPMI 1640 Medium supplemented with 20%, 20%, and 10% fetal bovine serum (FBS) respectively. OCI-Ly7 cells were cultured in Iscove's Modified Dulbecco’s Medium supplemented with 20% FBS. All cell lines were incubated at 37 °C in a 5% CO_2_ incubator. SHP-1 agonists, SC-43 and SC-60, were synthesized, and its quality was evaluated as described in a previous study (Liu et al. 2017a, 2013). For cell-based studies, SHP-1 agonists were dissolved in dimethyl sulfoxide (DMSO) and then added to the cells in medium containing 5% FBS. The Myc-DDK-tagged Lyn expression construct and pCMV6-Entry plasmids were purchased from OriGene (Rockville, MD, USA). DLBCL cells were transiently transfected with the PolyJet transfection reagent (SignaGen Laboratories, Frederick, MD, USA) following to manufacturer’s instructions. To knockdown endogenous SHP-1, cells were transfected with siRNAs against *PTPN6* (L-009778-00) or control (D-001810-10) (final concentration of 25 μM) for 72 h using DharmaFECT 1 Transfection Reagent (T-2001-01) according to manufacturer's instructions (Dharmacon, Chicago, IL, USA).

### Xenograft tumor growth

The animal study was approved by the Institutional Animal Care and Use Committee of Taipei Veterans General Hospital. All experimental procedures using these mice were performed in accordance with protocols approved by the Institutional Animal Care and Use Committee of Taipei Veterans General Hospital (IACUC No. 2016-202). Female NCr athymic nude mice (5–7 weeks of age) were purchased from the National Laboratory Animal Center (Taipei, Taiwan) and maintained in an SPF-environment. Each mouse was inoculated subcutaneously in the dorsal flank with 5 × 10^6^ U2932 cells suspended in 100 μl of a 1:1 mixture of phosphate-buffered saline and Matrigel (BD Biosciences, Bedford, MA, USA) under isoflurane anesthesia. Tumors were measured using calipers and their volumes calculated using a standard formula (width^2^ × length × 0.52). When tumors reached 200 mm^3^, mice were administered with SC-43 (10 and 30 mg/kg), ibrutinib (12.5 and 25 mg/kg) or vehicle orally thrice per week.

### SHP-1 phosphatase activity

Briefly, the tumor homogenates were incubated with anti-SHP-1 antibody in immunoprecipitation buffer (20 mM of Tris–HCl [pH 7.5], 150 mM of NaCl, 1 mM of ethylenediaminetetraacetic acid, 1% NP-40, and 1% sodium deoxycholate) overnight. Protein G-Sepharose 4 Fast flow (GE Healthcare Bio-Science, Township, NJ) was added to each sample, followed by incubation for 3 h at 4 °C with rotation and then assayed for phosphatase activity by RediPlate 96 EnzChek Tyrosine Phosphatase Assay Kit (Molecular Probes, Invitrogen, Carlsbad, CA).

### Western blot analysis

As described previously (Liu et al. 2019), whole-cell extracts were prepared using RIPA buffer with a Halt Protease and Phosphatase Inhibitor Cocktail (Thermo Fisher Scientific, Waltham, MA, USA). The cell lysates were then analyzed by sodium dodecyl sulfate–polyacrylamide gel electrophoresis using antibodies (Additional file 1: Table S1).

### DLBCL microarray and immunohistochemical (IHC) staining

Human paraffin embedded tissue microarrays of DLBCL were purchased from US Biomax (LY1002 and LY1001b; US Biomax*,* Rockville*, *MD, USA). The slide was deparaffinized with xylene for 5 min, followed by two changes of xylene; the slides were then rehydrated. The slides were incubated with 3% H_2_O_2_ for 10 min to block peroxidase activity and subsequently incubated with blocking solution (2% FBS and 1% BSA) for 1 h. The primary antibody against SHP-1 (ab2020; Abcam, Cambridge, MA, USA) was used at 1:100 dilution for 1 h incubation at room temperature. The slides were washed three times with PBS and detected using the EnVision Detection Systems Peroxidase/DAB, Rabbit/Mouse kit (Agilent, Santa Clara, CA, USA). The slides were counterstained with hematoxylin and subsequently dehydrated and mounted. The IHC intensity of SHP-1 was determined independently by a qualified pathologist, and rated as a scale of 0 to 3 +, and an H-score of 0–300 based on percentage of cells stained at different intensities were assigned to each sample. Median value of positive IHC H-score was used as a cut-off at 41.25 for defining high versus low expression of SHP-1.

### In silico survival analysis with public open databases

SHP-1 transcript expression data downloaded from Gene Expression Omnibus database (GSE57611 and GSE11318) were analyzed. To assess significantly enriched pathways by comparing SHP-1 high expression with low expression DLBCL, gene sets in the Kyoto Encyclopedia of Genes and Genomes (KEGG) were used for Gene Set Enrichment Analysis (GSEA). A *P*-value < 0.05 and false discovery ratio (FDR) < 0.25 were considered as statistical significance.

### Cell viability and apoptosis determination

Cell viability of DLBCL cells treated with SC-43, ibrutinib or ruxolitinib for 72 h were assessed by 3-(4,5-dimethylthiazol-2-yl)-2, 5-diphenyltetrazolium bromide (MTT) assay. Ten microliters of MTT solution (final concentration 0.5 mg/mL) was added to each well and incubated at 37 °C for 3 h. The formazan crystals were then dissolved in 100 μl of DMSO and the absorbance was measured at 570 nm. Drug-induced apoptotic cell death was assessed using propidium iodide staining and analyzed by flow cytometry.

### Statistical analysis

Data are depicted as mean ± SD or SE. Statistical comparisons were based on nonparametric tests and statistical significance was defined as a *P*-value less than 0.05. All statistical analyses were performed using SPSS for Windows software, version 22.0 (SPSS, Chicago, IL, USA).

## Results

### SHP-1 agonist suppresses DLBCL xenograft tumor growth

**Fig. 1:**
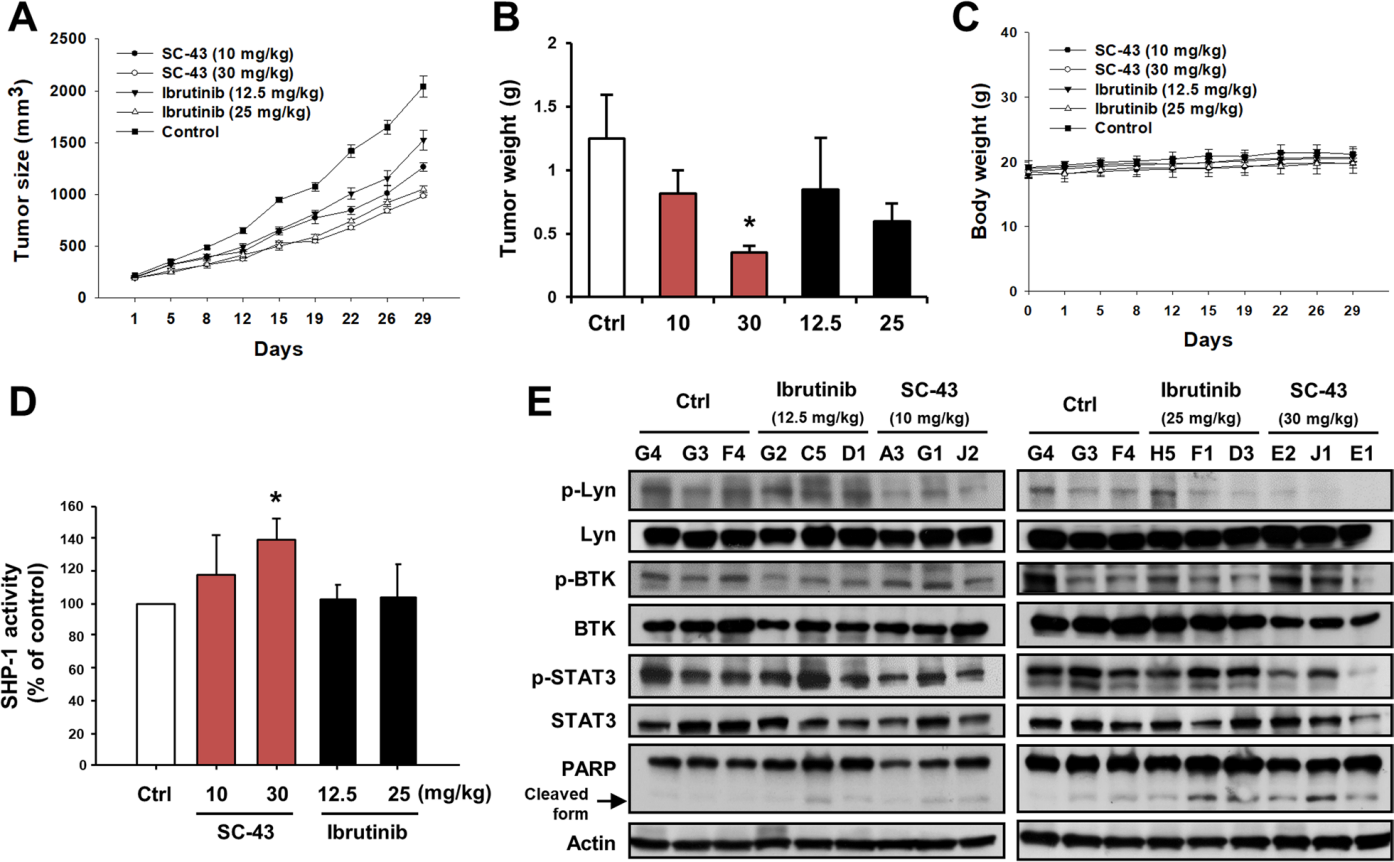
SHP-1 agonist suppresses tumor growth through SHP-1/Lyn pathway in vivo. **A**–**E** U2932 tumor-bearing mice (N = 4) were treated with vehicle, SC-43 (10 and 30 mg/kg) or ibrutinib (12.5 and 25 mg/kg) orally three times a week. Tumor growth (**A**), tumor weights **(B**), and body weights (**C**) of mice were measured. The SHP-1 activity and protein expression levels of tumors were analyzed by Tyrosine Phosphatase Assay (**D**) and Western blot analysis (**E**). Student’s *t*-test, *P < 0.05

Previously, we developed SHP-1 agonists, including SC-43 and SC-60, showed anti-cancer activities (Liu et al. 2017a). To assess the therapeutic potential of SHP-1 agonist and examine SHP-1/p-Lyn regulatory signaling in vivo, U2932 xenografted mice received vehicle, SC-43 or a selective BTK inhibitor ibrutinib. We observed that the mice receiving SC-43 (30 mg/kg) exhibited the potent tumor-suppressive effect (Fig. 1A and B). In addition, there were no apparent body weight loss or toxicity in the drug-treated mice in comparison with the control group (Fig. 1C). We checked whether SHP-1 agonist-mediated inhibition of tumor growth in U2932 cells depended on SHP-1/p-Lyn pathway. Compared with control groups, SC-43 increased SHP-1 activity (Fig. 1D). SC-43 dephosphorylated Lyn, BTK, and STAT3 and induced apoptosis in vivo (Fig. 1E). Moreover, another SHP-1 agonist SC-60 also reduced tumor growth and tumor weight without body weight loss (Additional file 1: Fig. S1A–C). Treatment of SC-60 elevated SHP-1 activity whereas suppressed p-Lyn and p-STAT3. Apoptosis also evidenced by PARP cleavage in U2932 xenografts (Additional file 1: Fig. S1D and E). These results indicated that SHP-1 agonist inhibited DLBCL tumor growth as well as inactivated Lyn and STAT3 signaling.

### SHP-1 is frequently expressed in DLBCL

**Fig. 2:**
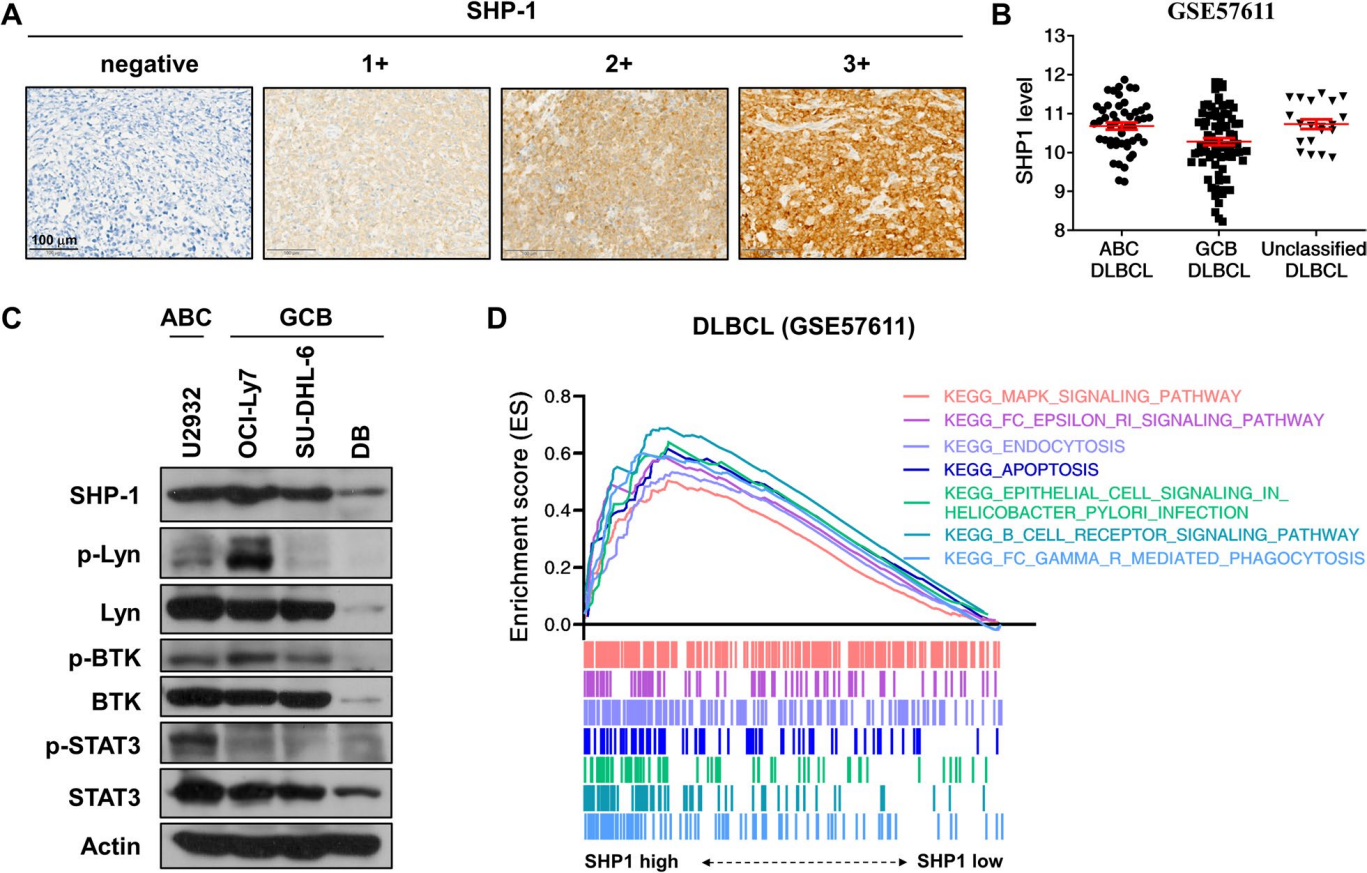
SHP expression and associated pathways in DLBCL patients. **A** Immunohistochemistry against SHP-1 was represented and scored (negative, 1 +, 2 +, and 3 +) on the DLBCL tissue microarrays (200× magnification times). **B** Expression of SHP-1 transcripts in DLBCL subtypes from Gene Expression Omnibus database were analyzed. **C** Whole-cell extracts of ABC DLBCL (U2932) and GCB DLBCL (OCI-Ly7, SU-DHL-6, and DB) cells were analyzed by Western blot analysis. **D** GSEA results showing the top 7 positive enrichment terms of the KEGG pathway gene sets

To evaluate the clinical relevance of SHP-1 in DLBCL, the expression of SHP-1 was examined by a DLBCL tissue microarray consisting of 150 DLBCL tumor samples (Additional file 1: Fig. S2A). A total of 114 (76%) of samples were positive for SHP-1 IHC staining, of which 57 (38%) samples had high expression of SHP-1. Representative SHP-1 expressions with IHC intensity scale from negative to 3 + were shown (Fig. 2A). The characteristics of the DLBCL tumors according to SHP-1 expression were examined. SHP-1 expression did not correlate with age, gender, and anatomic site (Additional file 1: Table S2). Data from Gene Expression Omnibus (GSE57611 and GSE11318) showed that ABC DLBCL had higher SHP-1 transcript level than GCB DLBCL (Fig. 2B and Additional file 1: Fig. S2B). We then assessed the endogenous SHP-1, Lyn, BTK, and STAT3 status of human DLBCL cell lines and found these proteins were differentially expressed in DLBCL cell lines (Fig. 2C). To explore the biological correlation of SHP-1 in DLBCL, the GSEA analysis of SHP-1 expression was performed based on DLBCL cohort (GSE57611). As shown in Fig. 2D, GSEA results indicated that SHP-1 high expression was associated with immune-related pathways. Notably, DLBCL with high SHP-1 expression was positively enriched in apoptosis pathway.

### SHP-1 agonist exerts anti-proliferative activity in DLBCL cell lines

**Fig. 3:**
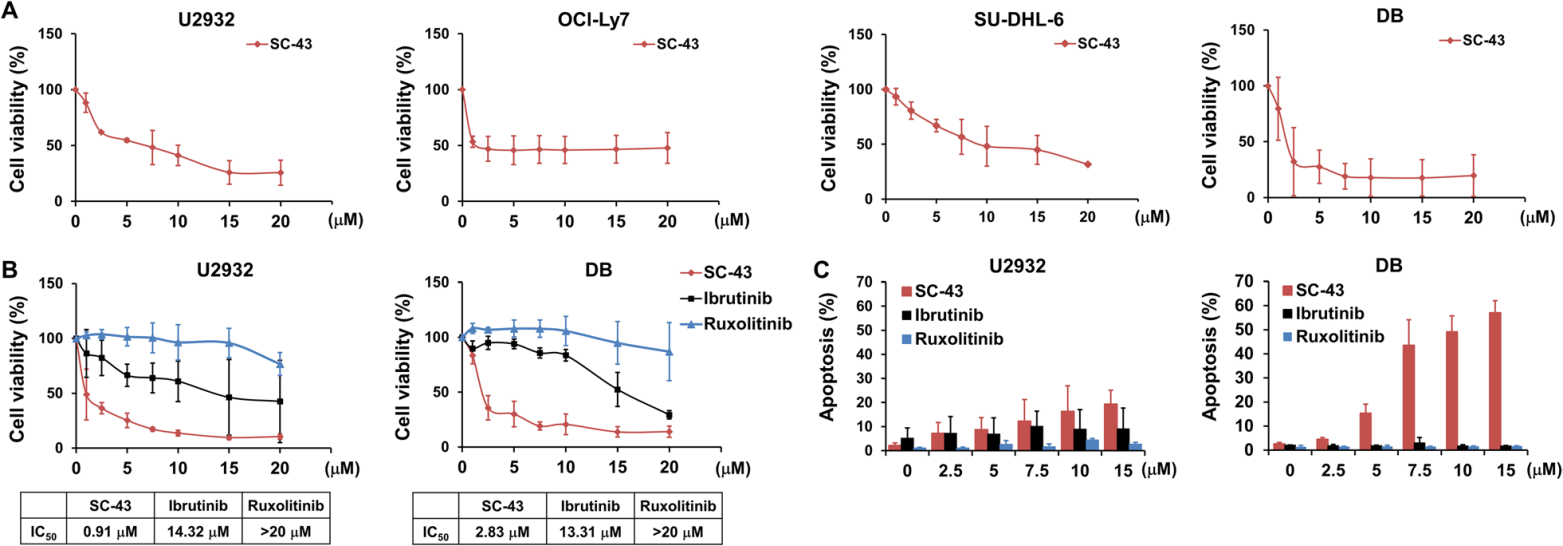
SHP-1 agonist shows anti-proliferation activity. **A** DLBCL cells treated with SC-43 at the indicated doses for 72 h were assessed by MTT assay. **B** U2932 and DB cells treated with SC-43, ibrutinib or ruxolitinib at the indicated doses for 72 h were assessed by MTT assay. **C** U2932 and DB cells treated with SC-43, ibrutinib or ruxolitinib at the indicated doses for 48 h were analyzed by flow cytometry. Data are representative of three independent experiments

To examine the effects of SHP-1 agonist in vitro, we used SC-43 to test the cytotoxic effect on DLBCL cells. Results showed that SC-43 inhibited cell viability of the four DLBCL cell lines (Fig. 3A). Previous studies demonstrated that SHP-1-suppressed STAT3 and led to cell apoptosis (Liu et al. 2017a). STAT3 activation promoted cell survival in ABC-type DLBCL (Ding et al. 2008), hence we evaluated the effects of SC-43, ibrutinib, and a JAK/STAT inhibitor ruxolitinib (Jakavi) in DLBCL cells. SC-43 significantly decreased cell viability and induced cell apoptosis of U2932 and DB cells. There was differential sensitivity to ibrutinib among U2932 and DB cells, while ruxolitinib exerted little effects in DLBCL cells in contrast to SC-43 (Fig. 3B and C). These data suggested that SHP-1 agonist exhibited anti-proliferative activity in vitro.

### SHP-1 agonist antagonizes BCR-related signaling in DLBCL cells

**Fig. 4:**
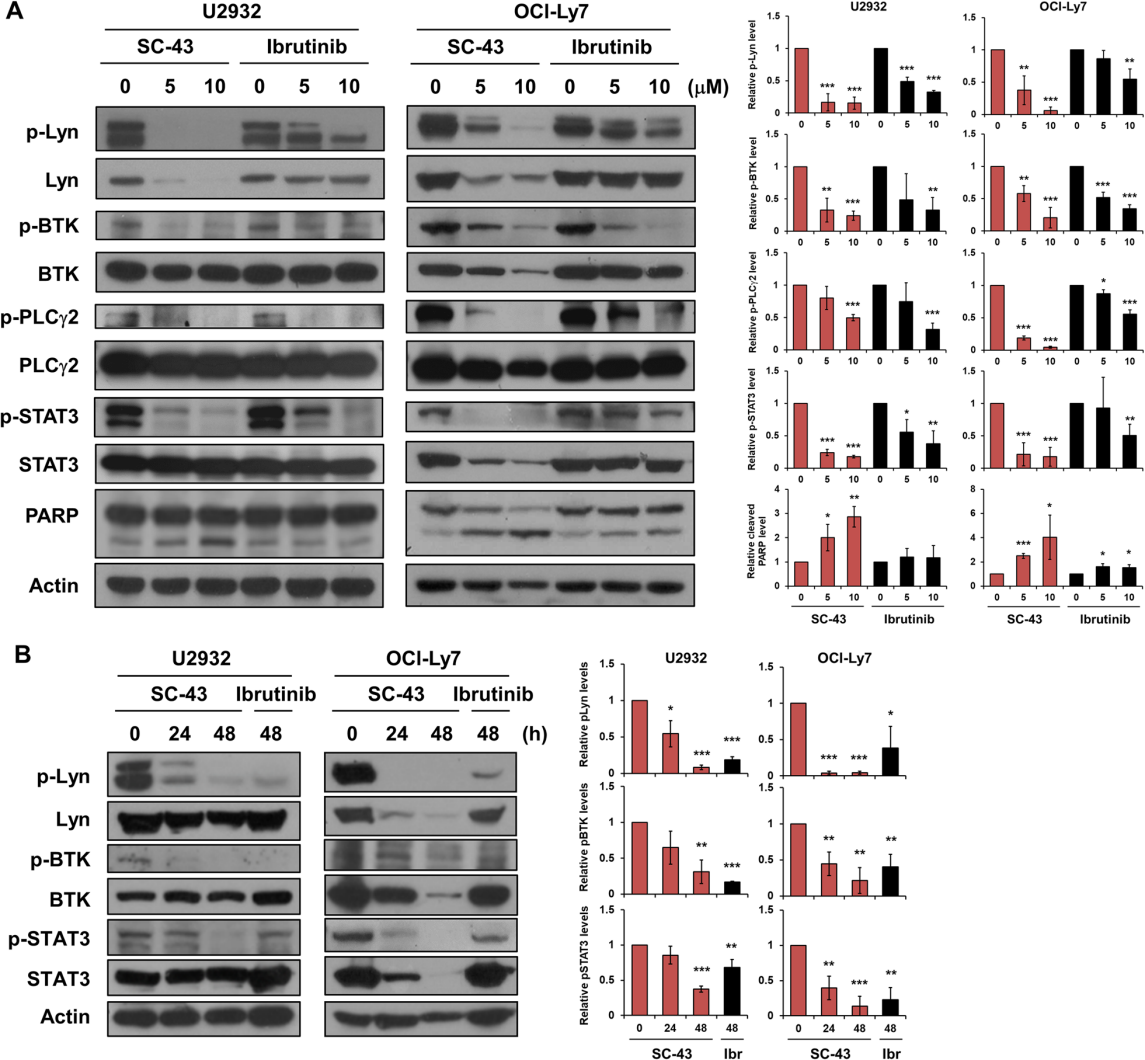
SHP-1 agonist inactivates Lyn signaling and elicits PAPR cleavage. **A** Whole-cell extracts of U2932 and OCI-Ly7 cells treated with SC-43 or ibrutinib at indicated doses for 24 h were analyzed by Western blot analysis (left). The quantitative results of blotting were shown (right). **B** Whole-cell extracts of U2932 and OCI-Ly7 cells treated with SC-43 or ibrutinib at 5 μM for indicated times were examined by Western blot analysis (left). The quantitative results of blotting were shown (right). Data are representative of three independent experiments. Student’s *t*-test, *P < 0.05; **P < 0.01; ***P < 0.001

We then examined the molecular events associated with SHP-1 agonist treatment in DLBCL cells. Treatment of SC-43 inactivated BCR signaling pathway, including Lyn, BTK, and PLCγ2, in a dose-dependent manner. Besides, SC-43 showed the potent effect on inhibition of pSTAT3 compared to ibrutinib. The cell apoptotic effect was validated by PARP cleavage (Fig. 4A). Moreover, SC-43 suppressed phosphorylation of Lyn, BTK, and STAT3 of DLBCL cells in a time-dependent manner (Fig. 4B). These results suggested that SHP-1 agonist effectively repressed BCR-related signaling.

### SHP-1 agonist induces cell apoptosis via Lyn inactivation

**Fig. 5:**
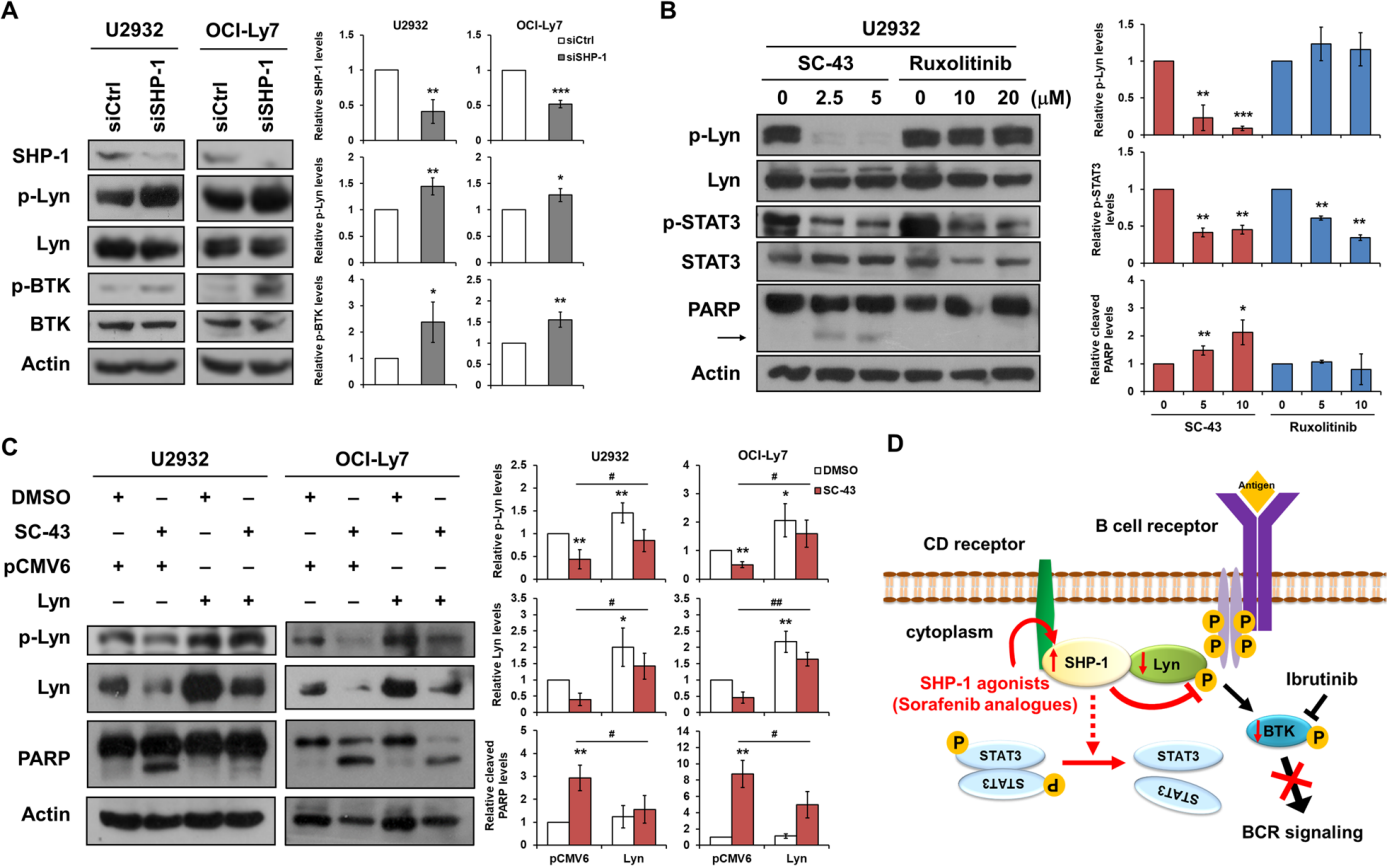
SHP-1 agonist induces cell apoptosis through Lyn. **A** Whole-cell extracts of U2932 and OCI-Ly7 transfected with siRNAs against SHP-1 or control were assessed by Western blot analysis (left). The quantitative results of blotting were shown (right). **B** U2932 cells treated with SC-43 or ruxolitinib at indicated doses for 24 h were examined by Western blot analysis (left). The quantitative results of blotting were shown (right). **C** U2932 and OCI-Ly7 cells were transfected with Lyn-expressing or control plasmids for 48 h. The transfected cells were further treated with 10 μM SC-43 or DMSO for 24 h and examined by Western blot analysis (left). The quantitative results of blotting were shown (right). **D** In the present study, our data indicated that SHP-1 agonists (sorafenib analogues such as SC-43 and SC-60) enhanced SHP-1 activity and further reduced phosphorylation of Lyn and BTK. Dephosphorylation of Lyn and BTK inhibited cell survival signaling leading to cell apoptosis. In addition, SHP-1 agonists also dephosphorylated STAT3 as previously reported which might partly contribute to cell growth inhibition. Data are representative of three independent experiments. Student’s *t*-test, *P < 0.05; **P < 0.01; ***P < 0.001; ^#^*P* < 0.05; ^##^*P* < 0.01

To further validate the effect of SHP-1 in BCR signaling, the siRNA-mediated knockdown of endogenous SHP-1 was performed. Results revealed that knockdown of SHP-1 increased phosphorylation of Lyn and BTK (Fig. 5A). We observed SHP-1 agonists effectively reduced the phosphorylation of Lyn and STAT3 to contrast with ruxolitinib was unable to downregulate p-Lyn (Fig. 5B, Additional file 1: Fig. S3A, and S3B). Consistent with the results shown in Fig. 3C, PARP cleavage was induced by SC-43 but not ruxolitinib (Fig. 5B). We next examined whether the inactivation of Lyn was crucial to the SHP-1 agonist-induced apoptosis in DLBCL cells. We transfected U2932 and OCI-Ly7 cells with exogenous Lyn and found that SC-43 induced PAPR cleavage was attenuated (Fig. 5C). Likewise, treatment of SC-60 induced PARP cleavage was also repressed following Lyn overexpression (Additional file 1: Fig. S3C). Taken together, these results demonstrated that SHP-1 agonist induced cell apoptosis through SHP-1/p-Lyn pathway in DLBCL cells.

## Discussion

The clinical significance of SHP-1 in cancers remains to be elucidated. Some studies have shown decreased SHP-1 expression was associated with poor outcome in prostate cancer (Tassidis et al. 2010), colorectal cancer (Fan et al. 2016), and triple-negative breast cancer (Liu et al. 2017b, c), whereas other studies demonstrated contrarily that increased SHP-1 expression is associated with recurrence in nasopharyngeal carcinoma (Peng et al. 2014) or aggressiveness in breast cancer (Insabato et al. 2009). Several studies have also explored the role of SHP-1 expression in lymphoma (Kossev et al. 2001; Leon et al. 2002; Oka et al. 2001; Witkiewicz et al. 2007; Zhang et al. 2000). Kossev et al. demonstrated that B lymphocytes in follicle germinal centers do not express SHP-1, whereas normal B cells in mantle and marginal zones or interfollicular B lymphocytes and plasma cells showed strong SHP-1 expression (Kossev et al. 2001). Similarly, Oka et al. analyzed SHP-1 expression by IHC in various kinds of malignant lymphomas including DLBCL and showed that more than 95% of malignant lymphomas were negative for SHP-1 expression (Oka et al. 2001). In contrast, we found 38% of DLBCL tumors express high SHP-1 expressions. However, considering there is a relative paucity of literature on SHP-1 expression in clinical patient samples with DLBCL, more studies are needed to better define the clinical roles of SHP-1 in DLBCL. In this study, we investigated the biological role and potential therapeutic implication of SHP-1 in DLBCL. SHP-1 agonists increased SHP-1 activity, whereas downregulated Lyn signaling*.* We demonstrated that SHP-1 was frequently (76%) expressed in various intensities in DLBCL tumors. SHP-1 agonist decreased BCR signaling by inhibiting p-Lyn, which led to apoptosis (Fig. 5D).

Somani et al. demonstrated that Lyn phosphorylation/dephosphorylation as a possible mechanism by which SHP-1 exerts its influence on CD19 tyrosine phosphorylation and its inhibitory effect on BCR signaling (Somani et al. 2001). Previous studies have consistently shown that SHP-1 agonists induce apoptosis via the SHP-1/p-STAT3 signaling axis in various cancer cells including hepatocellular carcinoma cells, breast cancer cells, and colorectal cancer cells (Liu et al. 2017a, 2017c; Chao et al. 2016; Fan et al. 2015). In the current study, we noticed that the JAK/STAT inhibitor ruxolitinib exerted little effects on cell viability and apoptosis in DLBCL cells in contrast to SC-43. The finding that SHP-1 agonists and ruxolitinib suppressed p-STAT3, whereas only SHP-1 agonists effectively suppressed p-Lyn and induced PARP cleavage suggesting SHP-1/p-Lyn axis might play a more important role in mediating the apoptosis effects in DLBCL cells, and supported the role of BCR signaling in DLBCL. Moreover, Wang et al. reported that B cell signaling activated STAT3 via Lyn in a JAK1/2-independent manner (Wang et al. 2007). It is also possible that SHP-1 agonist suppressed STAT3 activation through Lyn inhibition. In other words, although p-STAT3 inhibition may induce apoptosis, the suppression of p-Lyn may be a more important molecular determinant in apoptosis induction in DLBCL cells.

Indeed, prior studies have demonstrated that ABC-like DLBCL are more sensitive to ibrutinib, comparing to non-ABC like DLBCL (Wilson et al. 2015, 2021; Davis et al. 2010; Xue et al. 2020; Mondello and Ansell 2021). A meta-analysis has reported a pooled overall response (OR) of 41.6% for ibrutinib monotherapy and a pooled OR of 72.0% for combinational ibrutinib and rituximab-based therapy in patients with DLBCL. The pooled OR was reported as 64.2% in patients with non-GCB DLBCL (Hou et al. 2020). Nevertheless, the clinical data for ibrutinib monotherapy suggested its preferential efficacy toward non-GCB DLBCL and there is still unmet need in DLBCL. Mutations in *MYD88*, *PLCγ2*, *CARD11*, and *TNFAIP3* contribute to acquire resistance to ibrutinib (Wilson et al. 2015; George et al. 2020). U2932 cell line is probably attributed to the *TNFAIP3* mutation that confers resistance to BTK-targeting agents (George et al. 2020; Paul et al. 2017). However, our results showed that treatment with higher dose of ibrutinib (25 mg/kg) exhibited some anti-cancer activity in U2932 tumor-bearing mice. Previous studies also showed that U2932 xenografts displayed a reduction in tumor-background ratios following treatment with 25 mg/kg ibrutinib compared to controls (Jacobs et al. 2017). We observed that the levels of p-Lyn were decreased in 25 mg/kg ibrutinib treated xenografts (Fig. 1E). The tumor inhibitory effects of higher doses of ibrutinib might results from inhibition of signal pathways other than BTK. Importantly, our preclinical data showed that both ABC and GCB-like cells can be sensitive to SHP-1 agonists via the SHP-1/p-Lyn axis. More studies are needed to see whether there is differential anti-cancer activity via the SHP-1/p-Lyn axis among ABC and GCB-like DLBCL.

## Conclusions

In summary, our data further strengthens this notion by application of SHP-1 agonists which increase SHP-1 activities. Treatment with SHP-1 agonists to target SHP-1/p-Lyn axis demonstrate therapeutic potential in DLBCL in both in vivo and in vitro models.

## Supplementary Information

**Additional file 1:** **Table S1**. List of antibodies used for Western blot analysis. **Table S2**. Characteristics of tissue microarray of tumors from patients with diffuse large B cell lymphoma. **Figure S1**. SHP-1 agonist SC-60 suppresses tumor growth through SHP-1/p-Lyn pathway in vivo. **Figure S2**. Expressions of SHP-1 protein and transcript in DLBCL. **Figure S3**. SHP-1 agonist induces cell apoptosis through Lyn inhibition.

## Data Availability

The datasets generated and/or analysed during the current study are available in the Gene Expression Omnibus repository, GSE57611 and GSE11318.

## Abbreviations

DLBCL: Diffuse large B-cell lymphoma
BCR: B cell receptor
BTK: Bruton’s tyrosine kinase
NHL: Non-Hodgkin lymphoma
GCB: Germinal center B-cell
ABC: Activated B-cell
FBS: Fetal bovine serum
DMSO: Dimethyl sulfoxide
IHC: Immunohistochemical
KEGG: Kyoto encyclopedia of genes and genomes
GSEA: Gene set enrichment analysis
MTT: 3-(4,5-Dimethylthiazol-2-yl)-2, 5-diphenyltetrazolium bromide
