# Supplementary material for: Interfering B cell receptor signaling via SHP-1/p-Lyn axis shows therapeutic potential in diffuse large B-cell lymphoma
Source: Mol Med. 2022 Aug 8;28:93. doi: 10.1186/s10020-022-00518-0 (PMC9358803; doi:10.1186/s10020-022-00518-0)
Supplement: Supplementary file 1 — Additional file 1: Table S1. List of antibodies used for Western blot analysis. Table S2. Characteristics of tissue microarray of tumors from patients with diffuse large B cell lymphoma. Figure S1. SHP-1 agonist SC-60 suppresses tumor growth through SHP-1/p-Lyn pathway in vivo. Figure S2. Expressions of SHP-1 protein and transcript in DLBCL. Figure S3. SHP-1 agonist induces cell apoptosis through Lyn inhibition. [file 10020_2022_518_MOESM1_ESM.docx]

# Table S1. List of antibodies used for Western blot analysis

| **Primary antibodies** | **Dilution** | **Supplier** | **Reference** |
| --- | --- | --- | --- |
| p-Lyn | 1:1000 | abcam | ab40660 |
| Lyn | 1:1000 | Cell Signaling Technology | #2732 |
| p-BTK | 1:500 | Cell Signaling Technology | #5082 |
| BTK | 1:1000 | Cell Signaling Technology | #3533 |
| p-PLCγ2 | 1:500 | Cell Signaling Technology | #3874 |
| PLCγ2 | 1:1000 | Cell Signaling Technology | #3872 |
| p-STAT3 | 1:500 | Cell Signaling Technology | #9145 |
| STAT3 | 1:1000 | Cell Signaling Technology | #4904 |
| PARP | 1:2000 | Cell Signaling Technology | #9532 |
| SHP-1 | 1:1000 | Cell Signaling Technology | #3759 |
| beta Actin | 1:1000 | abcam | ab6276 |

# Table S2. Characteristics of tissue microarray of tumors from patients with diffuse large B cell lymphoma (n=150)

| **Characteristics** |  | **SHP-1 expression** | | | ***P* value** |
| --- | --- | --- | --- | --- | --- |
|  |  | **Negative  (*n* = 36)** | **Low  (n = 57)** | **High  (*n* = 57)** |  |
| Age | | | | | |
| ≤65 | | 25 (69.4) | 37 (64.9) | 41 (71.9) | 0.717 |
| >65 | | 11 (30.6) | 20 (35.1) | 16 (28.1) |  |
| Gender | | | | | |
| Male | | 27 (75.0) | 43 (75.4) | 46 (80.7) | 0.742 |
| Female | | 9 (25.0) | 14 (24.6) | 11 (19.3) |  |
| Organ | | | | | |
| Nodal | | 8 (22.2) | 24 (42.1) | 26 (45.6) | 0.062 |
| Extra nodal | | 28 (77.8) | 33 (57.9) | 31 (54.4) |  |

SHP-1 expression level is divided by median value of H-score (defined in Materials and Methods section).


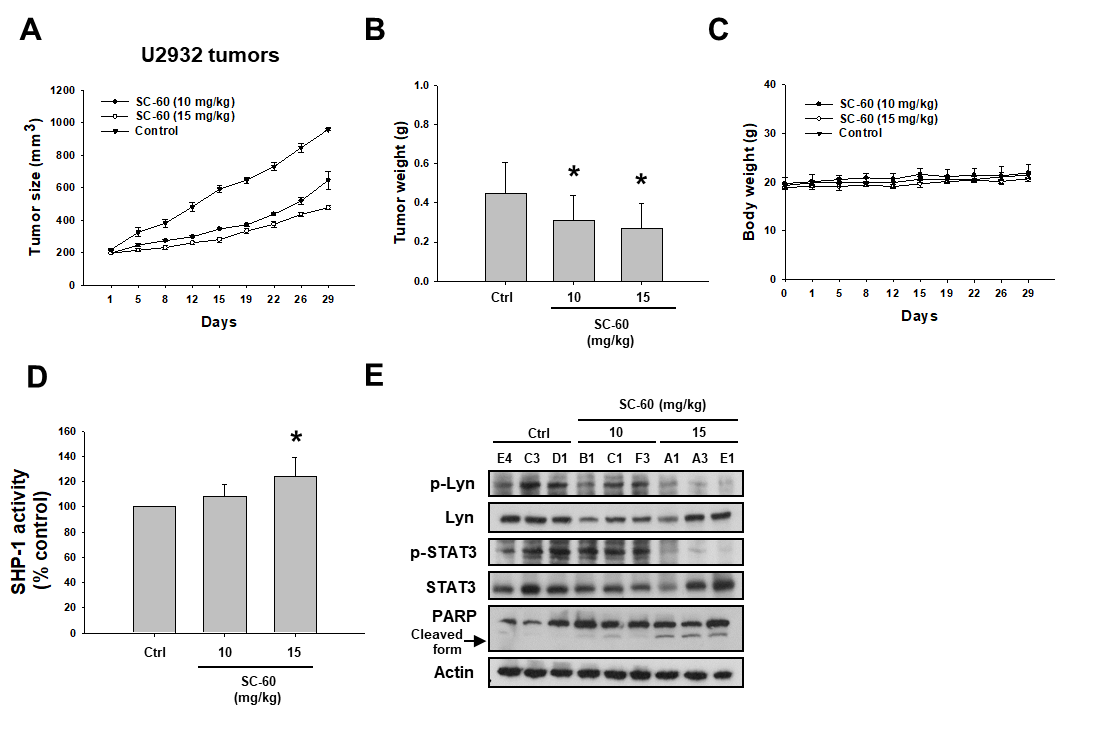


**Figure S1. SHP-1 agonist SC-60 suppresses tumor growth through SHP-1/p-Lyn pathway *in vivo*.**

(A-E) U2932 tumor-bearing mice were treated with vehicle or SC-60 (10 and 15 mg/kg) orally three times a week. Tumor growth (A), tumor weights (B), and body weights (C) of mice were measured. The SHP-1 activity and protein expression levels of tumors were analyzed by Tyrosine Phosphatase Assay (D) and Western blot analysis using anti-p-STAT3^Y705^, anti-STAT3, anti-p-Lyn^Y397^, anti-Lyn, anti-PARP, and anti-actin antibodies (E). Data of growth curve (n=4) are shown as mean ± SE. Data of tumor weight, body weight, and SHP-1 activity (n=4) are shown as mean ± SD. Student's *t*-test, *, *P* < 0.05.

**
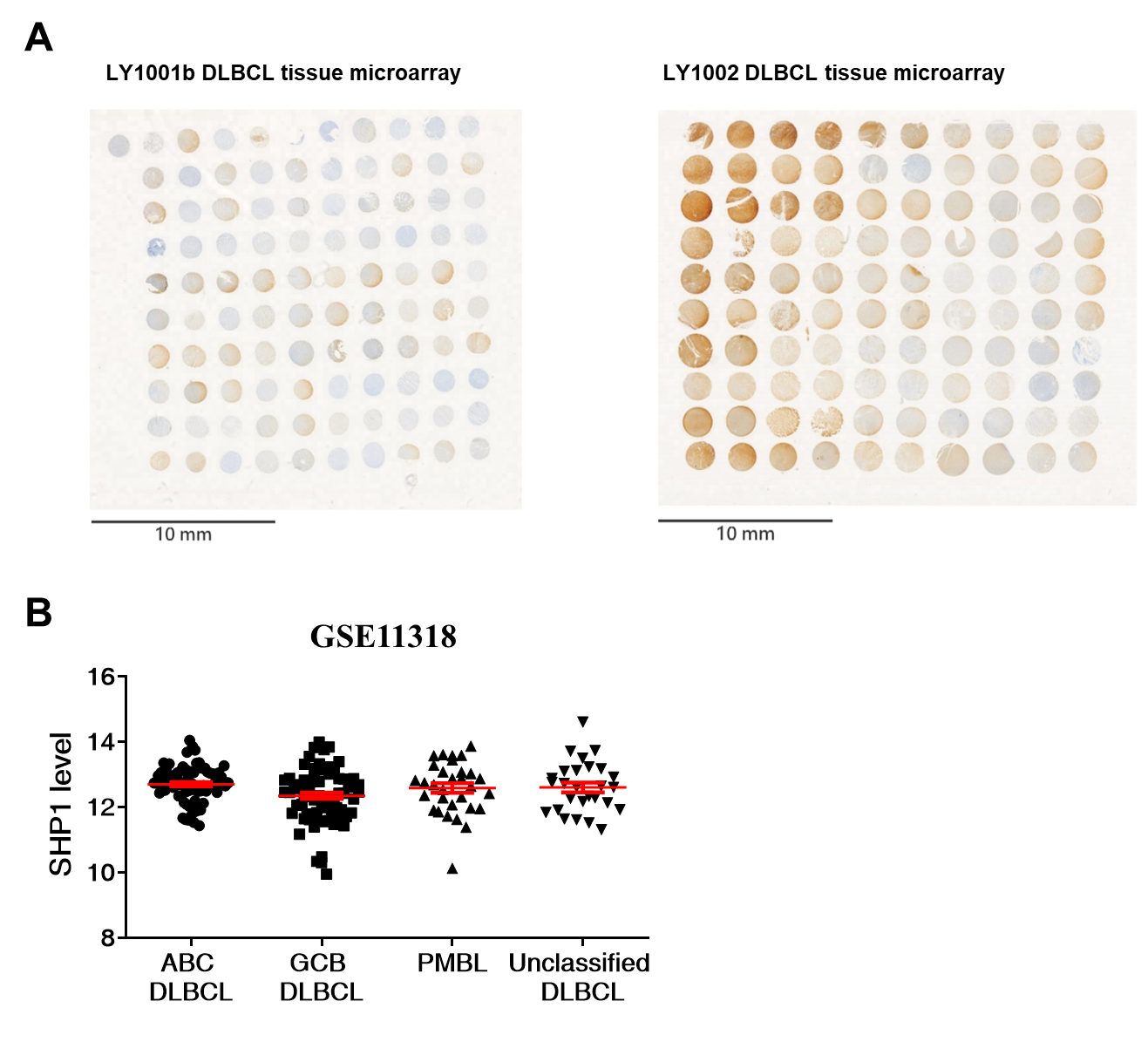
**

**Figure S2. Expressions of SHP-1 protein and transcript in DLBCL.**

(A) LY1001b DLBCL tissue microarray contained 100 cases/100 cores; LY1002 DLBCL tissue microarray contained 50 cases of DLBCL, duplicate core per case. (B) Expression of SHP-1 transcripts in DLBCL subtypes from Gene Expression Omnibus database were analyzed.

**
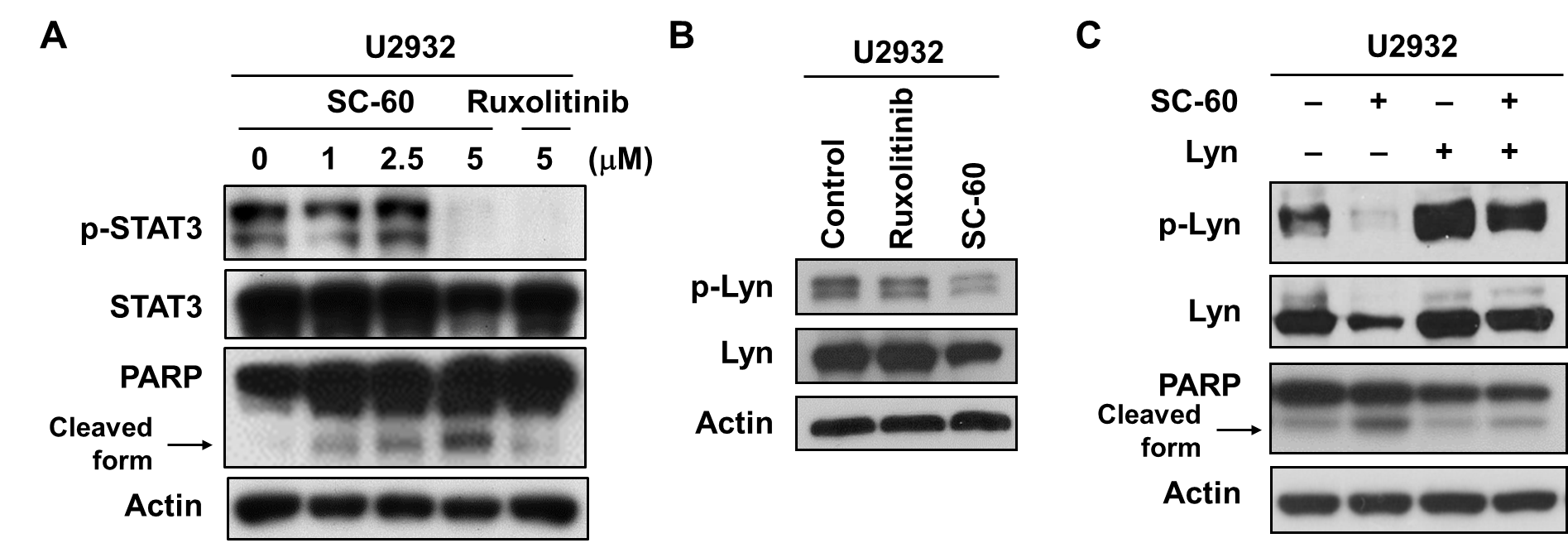
**

**Figure S3. SHP-1 agonist induces cell apoptosis through Lyn inhibition.**

(A) U2932 cells were treated with SC-60 or ruxolitinib at indicated doses for 24 h. The whole-cell extracts were analyzed by Western blot analysis using anti-p-STAT3^Y705^, anti-STAT3, anti-PARP, and anti-actin antibodies. (B) U2932 cells treated with SC-60 (5 μM), ruxolitinib (5 μM) or control for 24 h were examined by Western blot analysis using anti-p-Lyn^Y397^, anti-Lyn, and anti-actin antibodies. (C) U2932 cells transfected with Lyn-expressing plasmids or empty vector for 48 h were treated with SC-60 (2.5 μM) or vehicle for another 24 h. Whole-cell extracts were analyzed by Western blot analysis using anti-p-Lyn^Y397^, anti-Lyn, anti-PARP, and anti-actin antibodies.
